# A Novel Digital Closed Loop MEMS Accelerometer Utilizing a Charge Pump

**DOI:** 10.3390/s16030389

**Published:** 2016-03-18

**Authors:** Yixing Chu, Jingxin Dong, Baoyong Chi, Yunfeng Liu

**Affiliations:** 1 Department of Precision Instrument, Tsinghua University, Beijing 100084, China; chuyx11@mails.tsinghua.edu.cn (Y.C.), dongjx@mail.tsinghua.edu.cn (J.D.); 2 Institute of Microelectronics, Tsinghua University, Beijing 100084, China; chibylxc@tsinghua.edu.cn

**Keywords:** MEMS accelerometer, closed loop, sigma delta modulator, charge pump

## Abstract

This paper presents a novel digital closed loop microelectromechanical system (MEMS) accelerometer with the architecture and experimental evaluation. The complicated timing diagram or complex power supply in published articles are circumvented by using a charge pump system of adjustable output voltage fabricated in a 2P4M 0.35 µm complementary metal-oxide semiconductor (CMOS) process, therefore making it possible for interface circuits of MEMS accelerometers to be integrated on a single die on a large scale. The output bitstream of the sigma delta modulator is boosted by the charge pump system and then applied on the feedback comb fingers to form electrostatic forces so that the MEMS accelerometer can operate in a closed loop state. Test results agree with the theoretical formula nicely. The nonlinearity of the accelerometer within ±1 g is 0.222% and the long-term stability is about 774 µg.

## 1. Introduction

MEMS accelerometers are widely applied in numerous areas such as inertial navigation systems, smartphones, vehicle electronics, *etc.* [1,2]. Digital closed loop accelerometers have been intense areas of research focus because they require no analog to digital conversion when being used [3,4,5,6,7,8,9,10]. Interface circuits for MEMS accelerometers are of great advantage because of their small size, low power dissipation, nice nonlinearity and low thermal drift [9,10,11,12,13].

The displacement of the proof mass is sensed by the readout circuit, and electrostatic forces are applied accordingly to keep the proof mass at its zero position. The nonlinearity in the readout circuits can be eliminated due to the infinite static gain in the forward path of closed loop MEMS accelerometers. 

Traditional digital closed loop MEMS accelerometers contain a complicated timing diagram of sensing, reset and force feedback [8,9,10,11,12] or multiplex power supplies [8]. The system can be of vast complexity and huge in size. Furthermore, these architectures are not suitable for a single power supply standard CMOS process. Despite there being many commercial MEMS accelerometers [14,15,16,17,18] from different companies, none of these work in the digital closed loop configuration. LIS3LV02DL (ST) [14], ADXL103 (ADI) [15], MMA685X (Freescale) [16] and MXD6235M (MEMSIC) [17] are capable of digital out but they operate in the open loop mode. MS9000D (Colibrys) [18] is a closed loop accelerometer; however, its output is analog signal.

A novel digital closed loop method utilizing a charge pump [19,20,21], which can be easily realized, is proposed in this paper. This method makes it possible for interface circuits of MEMS accelerometers to be integrated on a single die on a large scale. It is effective for accelerometers of any range by adjusting the output of the charge pump correspondingly as well.

## 2. MEMS Accelerometer Utilizing Charge Pump

**Figure 1:**
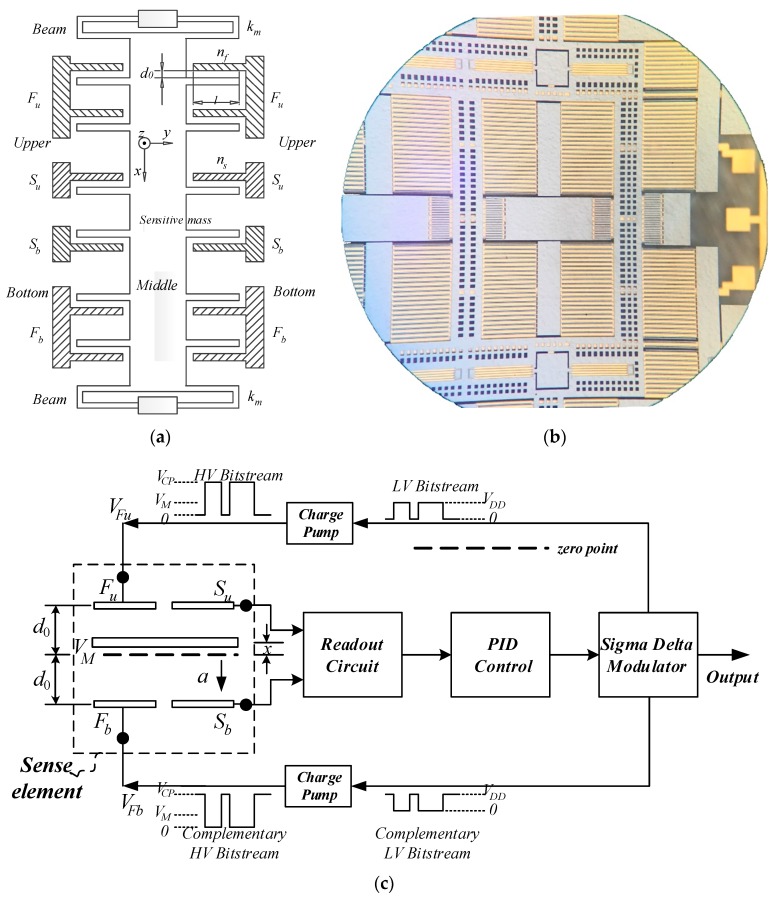
Architecture of the MEMS accelerometer: (**a**) Sense element used in MEMS accelerometer; (**b**) Microphoto of the sense element; (**c**) Scheme of the MEMS accelerometer system.

**Figure 2:**
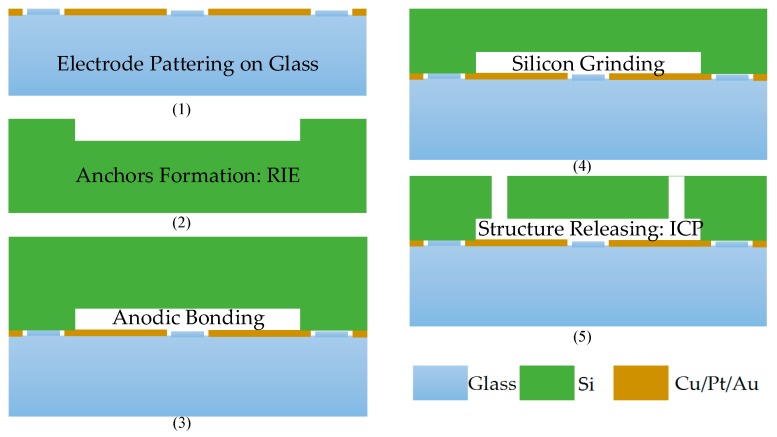
Fabrication process flow of the sense element.

The schematic and model of the sense element in Figure 1a is symmetric around both the horizontal and vertical axes as well as the center [22]. It is fabricated by Peking University using the SOG (silicon on glass) process (see microphoto in Figure 1b). Bottom and upper plates are fixed while the middle plate along with the proof mass moves as the input changes. Figure 2 shows the process flow to fabricate the sense element, and some important parameters are depicted in Table 1.

The differential capacitance ΔC of sensing comb fingers caused by the input acceleration is:

(1) $$\Delta C=C_{Su}-C_{Sb}=\frac{n_{s}\varepsilon_{0}\varepsilon A}{d_{0}-x}-\frac{n_{s}\varepsilon_{0}\varepsilon A}{d_{0}+x}=2C_{S0}[\frac{x}{d_{0}}+{\left(\frac{x}{d_{0}}\right)}^{3}+\cdots ]$$

where $n_{s}$ is the number of sensing comb fingers, ε is the relative dielectric constant, $\varepsilon_{0}$ is the absolute dielectric constant, A is the capacitance’s equivalent area and $d_{0}$ is the electrode gap with no input while $C_{S0}$ is the corresponding capacitance between the middle plate with the upper or bottom sense plate; x is the displacement of proof mass when the output of the readout circuit is zero. When feedback signals are applied on both the upper and bottom plates, the electrostatic force $F_{eu}$ generated by the upper plate ($F_{u}$) and $F_{eb}$ by the bottom plate ($F_{b}$) take effect simultaneously. The resultant electrostatic force $F_{e}$ applied on the proof mass can be calculated with the following equation:

(2) $$\begin{array}{l} F_{e}=\frac{1}{2}\frac{n_{f}\varepsilon_{0}\varepsilon A{\left(V_{Fu}-V_{M}\right)}^{2}}{{\left(d_{0}-x\right)}^{2}}-\frac{1}{2}\frac{n_{f}\varepsilon_{0}\varepsilon A{\left(V_{Fb}-V_{M}\right)}^{2}}{{\left(d_{0}+x\right)}^{2}}\\ =\frac{C_{F0}}{2d_{0}}\frac{[{(V_{Fu}-V_{M})}^{2}-{(V_{Fb}-V_{M})}^{2}]+2\frac{x}{d_{0}}[{(V_{Fu}-V_{M})}^{2}+{(V_{Fb}-V_{M})}^{2}]+{(\frac{x}{d_{0}})}^{2}[{(V_{Fu}-V_{M})}^{2}-{(V_{Fb}-V_{M})}^{2}]}{{(1-{(\frac{x}{d_{0}})}^{2})}^{2}} \end{array}$$

where $n_{f}$ is the number of feedback comb fingers, $V_{Fu}$ is the high voltage feedback signal generated by the charge pump applied on the upper feedback plate, and $V_{Fb}$ is the complementary one on the bottom feedback plate. $V_{M}$ is the excitation signal applied on the proof mass to detect the differential capacitance ΔC. 

As is shown in Figure 1c, the output voltage of the readout circuit, which is proportional to ΔC, is compensated by the proportional–integral–derivative (PID) controller. The compensated signal is then converted into the bitstream by the sigma delta modulator [23,24,25]. Then the bitstream can be converted into a digital signal by the digital decimation filter. When the output bitstream is 1, $V_{Fu}$ equals the output of the charge pump $V_{CP}$ and $V_{Fb}$ is connected to the ground. On the contrary, $V_{Fu}$ is connected to the ground and $V_{Fb}$ equals $V_{CP}$. When the MEMS accelerometer works in the closed loop configuration, $x\ll d_{0}$ so high-order terms of $x/d_{0}$ can be ignored. Therefore,

(3) $$F_{e}=\{\begin{array}{c} =\frac{C_{F0}}{2d_{0}}[(V_{CP}^{2}-2V_{CP}V_{M})+2(V_{CP}^{2}-2V_{Cp}V_{M}+2V_{M}^{2})\frac{x}{d_{0}}],\mathrm{bitstream}=1\\ =\frac{C_{F0}}{2d_{0}}[-(V_{CP}^{2}-2V_{CP}V_{M})+2(V_{CP}^{2}-2V_{Cp}V_{M}+2V_{M}^{2})\frac{x}{d_{0}}],\mathrm{bitstream}=0 \end{array}$$

The frequency of the bitstream is far higher than the natural frequency of the sense element, and only the low frequency component of $F_{e}$ is taken into account. Assuming the percentage of “1”s in the bitstream is D, then the DC component of the bitstream, *i.e.*, the digital output, is $V_{out}=DV_{DD}$, where $V_{DD}$ is the supply voltage of the circuit. The low frequency component of $F_{e}$ can be written as:

(4) $$F_{e}=\frac{C_{F0}}{2d_{0}}(V_{CP}^{2}-2V_{CP}V_{M})(2D-1)+\frac{C_{F0}}{d_{0}}(V_{CP}^{2}-2V_{Cp}V_{M}+2V_{M}^{2})\frac{x}{d_{0}}$$

In the static closed loop state, the electrostatic force $F_{e}$ balances the sum of other forces on the proof mass [26,27], namely

(5) $$F_{e}+({K\;}_{m}(x_{0}-x)-ma)=0$$

where $x_{0}$ is the initial deviation from the zero point of the proof mass when no stress is applied on the feedback beam, $K_{m}$ is the stiffness of the feedback beams and m is the mass of the proof mass. Substituting a=±1 g into Equations (4) and (5), after subtraction, Equation (6) of the scale factor of the MEMS accelerometer can be obtained as following:

(6) $$K_{1}=\frac{D_{+1g}-D_{-1g}}{2}V_{DD}=\frac{mgd_{0}V_{DD}}{C_{F0}}\frac{1}{V_{CP}^{2}-2V_{CP}V_{M}}$$

It can be concluded from Equation (6) that the scale factor is inversely proportional to the parabolic function of $V_{CP}$. The proposed digital closed loop MEMS accelerometer works only if $V_{CP}>2V_{M}$. A high input range can be achieved by increasing $V_{CP}$, *i.e.*, the output of the charge pump.

The variation of $F_{e}$ caused by the ripple of the charge pump can be calculated with:

(7) $$\Delta F_{e}{|}_{V_{CP}}=\frac{\partial F_{e}}{\partial V_{CP}}\Delta V_{CP}=\frac{C_{F0}}{d_{0}}(V_{CP}-V_{M})(2D-1)\Delta V_{CP}$$

From Equations (6) and (7), we can get:

(8) $$\frac{\Delta F_{e}{|}_{V_{CP}}}{mg}=\frac{V_{DD}}{K_{1}(V_{CP}^{2}-2V_{CP}V_{M})}(V_{CP}-V_{M})(2\frac{V_{out}}{V_{DD}}-1)\Delta V_{CP}$$

$F_{e}$ will be stable if the non-ideal behaviors such as ripple and drift of $V_{CP}$ are small enough. As Equation (6) indicates, $K_{1}\left(V_{CP}^{2}-2V_{M}V_{CP}\right)$ is a constant value, so we need to reduce the bias of the output voltage $V_{out}-\;V_{DD}/2$ and make $V_{M}-V_{CP}$ as small as possible to reduce the variation of $F_{e}$.

## 3. Charge Pump System

**Figure 3:**
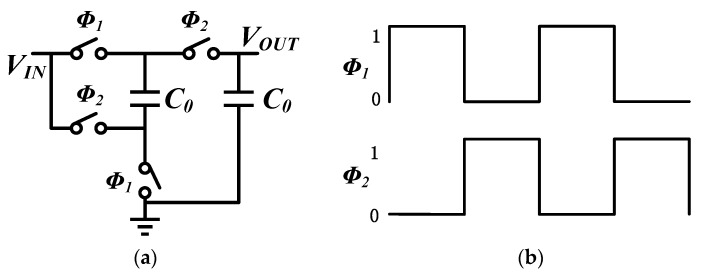
Makowski charge pump: (**a**) Schematic of 2X Makowski charge pump; (**b**) Non-overlapping clock.

**Figure 4:**
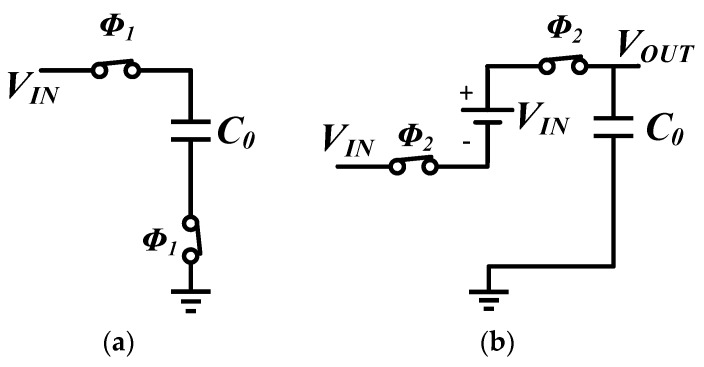
Equivalent circuit of Makowski charge pump: (**a**) during phase $\phi_{1}$; (**b**) during phase $\phi_{2}$.

Figure 3a illustrates the schematic of the 2X Makowski charge pump. [19] The non-overlapping phases $\phi_{1}$ and $\phi_{2}$ which control the switch in the Makowski charge pump are as depicted in Figure 3b. Equivalent circuits during both phases are exhibited in Figure 4.

During $\phi_{1}$, the voltage across $C_{0}$ is charged to $V_{IN}$. The voltage across the capacitor remains $V_{IN}$ when switching to phase $\phi_{2}$, therefore $V_{OUT}$ is charged by $V_{IN}$ and the charge on the capacitor. The two-phase non-overlapping clock must ensure that the switches are operated in a “break before make” fashion; otherwise, there will be a short-circuit current during switching [20]. Assuming $R_{ON}$ is the on resistance of the switches and $I_{L}$ is the load current,$\;V_{OUT}$ can be deduced as following:

(9) $$V_{OUT}=2V_{IN}-8I_{L}R_{ON}$$

**Figure 5:**
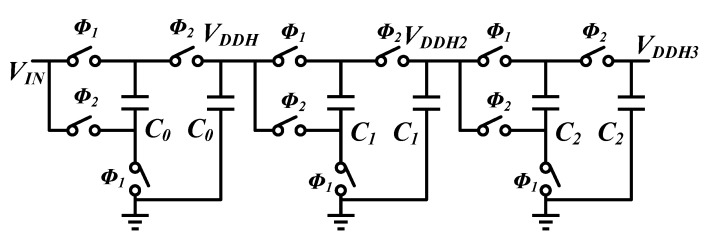
Cascade charge pump.

The output voltage is a little smaller than the theoretical twice the input because of the current leakage. Ripples can be seen in the output due to the switching. For a CMOS process, the supply voltage may be 5 V, 3.3 V, 1.8 V and so on. The output voltage of a 2X Makowski is far from what we need. A cascade charge pump using a single 5 V power supply (Figure 5) is proposed to get a high voltage of above 20 V.

**Figure 6:**
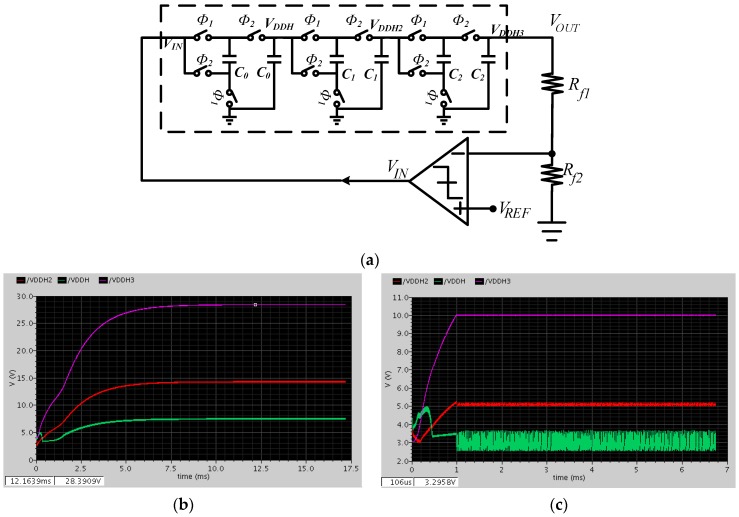
Charge pump: (**a**) Charge pump system with feedback control module; (**b**) Output of cascade charge pump (simulation); (**c**) Output of charge pump system with control module (simulation).

In order to suppress the ripple and maintain the output voltage, a feedback control module is adopted in Figure 6a [21]. A two-stage open loop comparator is used [28]. When V_OUT_·R_f2_/(R_f1_ + R_f2_) is smaller than $V_{ref}$, the output of the comparator ($V_{IN}$) is connected to the power supply and $V_{OUT}$ rises because of the charge pumping. On the contrary, $V_{IN}$ is connected to ground. As a result, $V_{OUT}$ will be maintained dynamically at a constant value:

(10) $$V_{OUT}=\frac{R_{f1}+R_{f2}}{R_{f2}}V_{ref}$$

Simulation results using Cadence Virtuoso are presented in Figure 6b,c. The output voltage of the output of the designed cascade charge pump can be pumped to as high as 28 V (Figure 6b), and the ripple is about 20 mV. The output of the first stage is only 7 V as the large load current is needed to drive the other stages. By using the feedback control module, the output voltage can be kept at a desired value such as 10 V (Figure 6c).

## 4. Results and Discussion

**Figure 7:**
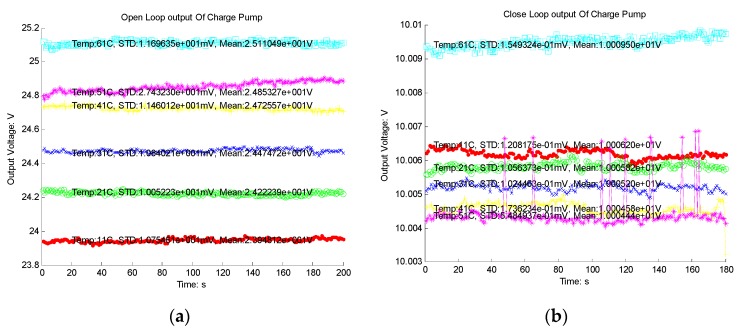
Charge pump: (**a**) Output of cascade charge pump; (**b**) Output of charge pump system with control module.

The charge pump system was designed and fabricated in a 0.35 µm HHGRACE high voltage CMOS process. Experimental results of the output of the designed cascade charge pump are presented in Figure 7a. The output voltage can be pumped to as high as 25 V, and the ripple is about 30 mV. However, the voltage value varies 4.48% when the temperature changes from 10 °C to 60 °C. By using the feedback control module, the output voltage can be kept at a desired value such as 10 V with a 0.2 mV ripple and 0.05% variation from 10 °C to 60 °C (Figure 7b). These results coincide with the simulation results very well.

**Figure 8:**
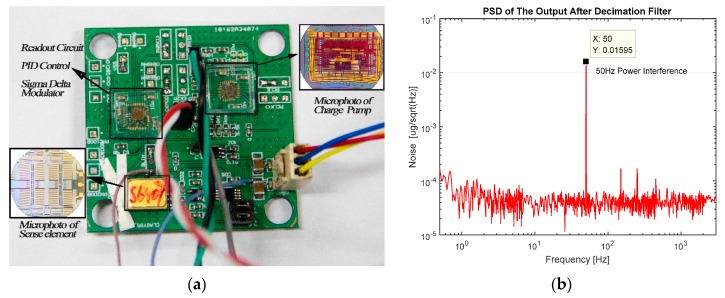
The MEMS accelerometer: (**a**) The laboratory prototype of MEMS accelerometer using charge pump; (**b**) PSD of the accelerometer’s output after decimation filter.

From Equation (6), we can conclude that the scale factor of the accelerometer is identically proportional to the inversion of $V_{CP}^{2}-2V_{M}V_{CP}$. Three sensors labeled S6401, S6407 and S6410 are used to form the proposed accelerometer system. Experiments are carried out while $V_{CP}$ varies orderly. Different value of $V_{M}$ such as 2.5 V and 1.25 V are adopted for these accelerometers. The laboratory prototype of the MEMS accelerometer using a charge pump (Figure 1c) is depicted in Figure 8a. PSD of the output after the decimation filter gathered using Agilent 35670 A shows a noise floor of about 30 µg/√Hz (Figure 8b).

**Figure 9:**
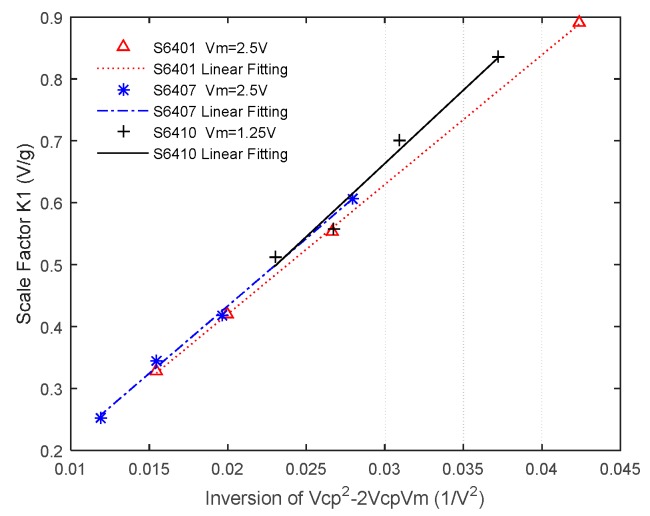
The relation between scale factor and the inversion of $V_{CP}^{2}-2V_{M}V_{CP}:$ For accelerometer S6401 and $V_{M}=2.5\;V$, the fitting function is y=20.9392x+0.0013; For accelerometer S6407 and $V_{M}=2.5\;V$, the fitting function is y=21.7927x−0.0026; For accelerometer S6410 and $V_{M}=1.25\;V$, the fitting function is y=23.6883x−0.0471.

Test results (Figure 9) and their linear fitting coincide the theoretical formula very well by using MATLAB polyfit. For different accelerometers and different values of $V_{M}$, the scale factor is always nearly proportional to the inversion of $V_{CP}^{2}-2V_{M}V_{CP}$.

**Figure 10:**
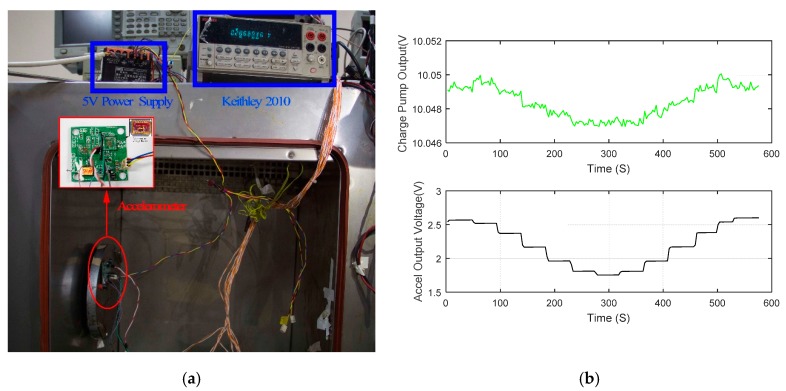
The 12-point tumble test: (**a**) The experimental setup; (**b**) The relation between $V_{CP}$ and the input acceleration.

**Figure 11:**
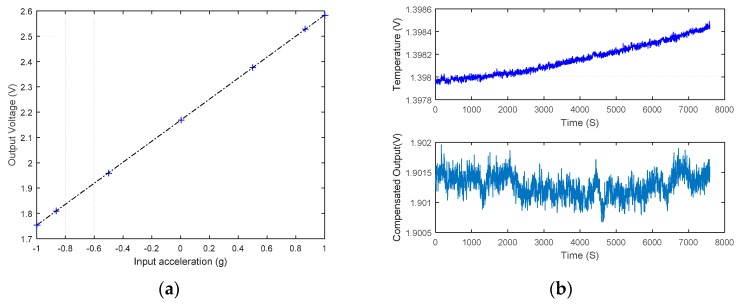
Test results of the accelerometer: (**a**) Relation between output and input acceleration; (**b**) Long-term stability of K0.

The experimental setup of the 12-point tumble test is depicted in Figure 10a. The output of the accelerometer is recorded every 30 degrees. Keitheley 2010 is used to collect data which is then saved and processed by using a computer. Twelve raw numbers acquired per rotation can be used to calculate the nonlinearity of accelerometer. As a component of the proposed accelerometer system, the output of the charge pump may change a little along with the input acceleration (see 3.1 mv Vpp of S6401 in Figure 10b). The same can be observed in other accelerometers. The dashed curve (see Figure 11a) using data from Figure 10 reveals the relation between the output of the accelerometer S6401 and the input acceleration within ±1 g. The nonlinearity of S6401 is 0.222% (when the input acceleration is −1 g). The 2 mV deviation of $V_{CP}$ at −1 g contributes a nonlinearity of 0.072% (32.6% of all) as Equation (8) reveals.

The output will drift when the temperature is changing, such as S6407 demonstrated in Figure 11b. The experiments were conducted in the evening so the output of the temperature-measuring circuit in the top subplot increases slowly. The raw data of the accelerometer’s output can be calibrated to diminish the influence of thermal drift thanks to the temperature-measuring circuit on the PCB. The long-term bias stability of the compensated output using temperature voltage is about 774 µg (see the bottom subplot of Figure 11b). The output of the charge pump system is adjusted to about 12 V, with a 178 µV standard deviation of compensated $V_{CP}$ which contributes 467 µg (60.3% of all) to the long-term bias stability using Equation (8). This is partly caused by the great deviation of system output (about 3 V) from 2.5 V.

From the experimental results, the ripple and thermal drift of the charge pump’s output restrict the performance of the new MEMS accelerometer greatly. Further work should focus on reducing the non-ideal behaviors of the charge pump system. A more accurate bandgap reference to generate $V_{ref}$, a higher frequency clock and a comparator of better performance may lead to more stable and low noise output of the charge pump system and, in turn, a more precise accelerometer.

## 5. Conclusions

The design and experiments of a new MEMS accelerometer using a charge pump are presented. Complicated timing diagrams of sensing, reset and force feedback or multiplex power supplies have been avoided. The new MEMS accelerometer can be integrated on a single die so that it can be small in size and suitable for many applications such as intelligent hardware and internet of things (IoT). Firstly, the architecture of the accelerometer is illuminated. The sensing and feedback theory are investigated, and the formula of the scale factor is deduced. Additionally, the charge pump’s implementation is described in detail. Then, the theory of the scale factor is validated based on the analysis of the experimental data; therefore, the accelerometer has a changeable scale factor associated with $V_{CP}$. Finally, test results show that the accelerometer has a nonlinearity of 0.222% and a bias stability of 774 μg. Analysis shows the output noise of the charge pump system restricts the performance of the accelerometer greatly.

Nonetheless, a high voltage CMOS process is needed to fabricate the charge pump. Compared to the normal process, a high voltage one has transistors of slower response and larger parasitical effect so the design of the circuits can be very demanding.

## Figures and Tables

**Table 1:** Parameters of the sense element used.

Parameter (Symbol)	Value	Parameter (Symbol)	Value
Sensitive mass (*m*)	0.57 mg	Total capacitance (*C_S_*_0_)	9.66 pF
Damping coefficient (*b*)	$10{.31\;\times \;10}^{-3}\text{ N}\cdot s/m$	Comb overlap length (*l*)	300 µm
Stiffness (*K_m_*)	150 N/m	Number of sensing comb fingers ($n_{s}$)	188
Comb spacing (*d*_0_)	3.1 µm	Number of feedback comb fingers ($n_{f}$)	188
